# Immunotherapy of Canine Leishmaniasis by Vaccination with Singlet Oxygen-Inactivated *Leishmania infantum*

**DOI:** 10.3390/vaccines14010062

**Published:** 2026-01-04

**Authors:** Laura Manna, Raffaele Corso, Bala K. Kolli, Namhee Kim, Kwang Poo Chang

**Affiliations:** 1 Dipartimento di Medicina Veterinaria e Produzioni Animali, Università di Napoli Federico II, Via F. Delpino 1, 80137 Napoli, Italy; laumanna@unina.it; 2 Dipartimento di Prevenzione, ASL NA1 Centro, Via Comunale del Principe 13A, 80145 NA1 Napoli, Italy; raffaelecorso@gmail.com; 3 Department of Microbiology/Immunology, Center for Cancer Cell Biology, Immunology and Infection, Chicago Medical School, Rosalind Franklin University of Medicine and Science (RFUMS), North Chicago, IL 60064, USA; balakolli@yahoo.com; 4 Center for Health Equity Research, Michael Reese Research and Education Foundation, Rosalind Franklin University, North Chicago, IL 60064, USA; namhee.kim@rosalindfranklin.edu

**Keywords:** canine leishmaniasis, *Leishmania infantum*, immunotherapy, chemo-immunotherapy, singlet oxygen-killed promastigotes, vaccine format, parasite load, clinical score

## Abstract

**Background**: Canine leishmaniasis is notoriously difficult to manage by chemotherapy alone, necessitating the consideration of supplemental or alternative treatment. Evidence is presented to support the feasibility of immunotherapy of diseased dogs through vaccination. **Methods**: The vaccine format used consisted of cultured promastigotes of *Leishmania infantum,* which were rapidly and completely killed by intracellularly generated singlet oxygen. A total of 33 owned dogs of different breeds and ages diagnosed positive for leishmaniasis were enrolled and divided into three groups for treatments as follows: (1) immunotherapy alone (9 dogs); (2) immunotherapy after chemotherapy (14 dogs); and (3) chemotherapy alone (10 dogs). All dogs in Groups 1 and 2 received intradermally three identical dosages of the vaccine format mentioned at the same schedules. The outcomes were assessed for one year at a post-treatment interval of 2–4 months by determining lymph node parasite loads and clinical scores based on established methodologies. **Results**: Spaghetti plots of the values for parasite loads obtained revealed that they scattered widely over time with a significant decline by 8–12 months post-treatment in all three groups. Sankey plots of clinical scores in stacked bars also showed that they followed erratic patterns of flow over time, albeit toward lower levels in all cases. Ordinal logistic regression analysis of clinical scores indicated that, while the odds for the emergence of severe clinical symptoms declined in all three groups, the lowest risk was associated with Group 2 dogs treated with immunotherapy after chemotherapy. The evidence presented thus suggests that immunotherapy of the diseased dogs with the vaccine format diminished their parasite loads and improved their clinical scores, especially when applied after chemotherapy. Dogs in Groups 1 and 2 that received immunotherapy, on average, lived twice as long as those in Group 3 that received chemotherapy alone. The risk of death estimated by analysis of the clinical scores using the Cox proportional hazard model was also found to be lower for Groups 1–2 dogs receiving immunotherapy than those in Group 3 receiving chemotherapy alone. **Conclusions**: Post-therapeutic survival time thus may be an additional parameter suitable to assess treatment efficacy by vaccination. In vitro approaches to mitigate some limitations of this study were proposed for future investigation.

## 1. Introduction

Canine visceral leishmaniasis caused by *Leishmania infantum* is a significant veterinary and public health problem in many endemic countries, including those in the Mediterranean basin. *Leishmania infantum* (syn. *L. chagasi*) is the etiological agent of both canine leishmaniasis (CanL) and zoonotic visceral leishmaniasis in children and immuno-compromised adults. In humans as well as in dogs, disease symptoms are severe and can be fatal if left untreated. Effective control of CanL is expected to reduce human infection by *L. infantum*, since dogs are its main reservoir [1,2]. There has been an increase in the incidence of CanL, calling attention to the urgent need for research not only for diagnosis and prevention but also for treatment of this complex disease [3].

Currently, chemotherapy is the mainstay in treating CanL with daily injections of meglumine antimoniate for about one month and allopurinol for life [4,5,6]. Meglumine antimoniate has anti-leishmanial properties and also enhances the phagocytic capacity of macrophages [7,8]. Allopurinol is leishmanostatic and is considered suitable for long-term administration to limit parasite loads, thereby potentially preventing disease relapses [8]. This combination chemotherapy has been reported to markedly decrease the parasite loads during the first 3 months [9]. In our previous study, we observed that chemotherapy rapidly decreased the parasite loads, concomitant with a significant clinical improvement, in the first month followed by a linear decrease in both over the subsequent nine months. In addition, we found a positive correlation between the clinical scores and blood pathological and serological events, e.g., the anti-*Leishmania* antibody titers. However, the incidence of disease recurrence is frequent 12 months after chemotherapy [5], especially when using miltefosine as an alternative to the antimonial [8]. There is an urgent need to develop new treatment strategies because the current chemotherapy is toxic, cumbersome to apply and invariably elicits drug-resistance [9].

An alternative approach to chemotherapy for treating CanL is immunotherapy, which has indeed been undertaken experimentally either alone or in combination with chemotherapy (immunochemotherapy) [10,11]. The antigen(s) used included in vitro cultured promastigotes after inactivation [12] and peptides or other molecular vaccines originally designed mostly for immunoprophylaxis of CanL (reviewed in [13]). Specifically detailed in this review are four licensed vaccines, among a list of unlicensed ones, i.e., Leish-Tech, Leishmune, CaniLeish and Letifend, containing *Leishmania* antigen A2, nucleoside hydrolase (NH36), purified excretory glycoproteins and nucleosomal histones, respectively, all used with Saponin/QA21 as the adjuvant. None of these vaccine formats has been considered as effective [14] to warrant their clinical use for immunotherapy of CanL on a routine basis. Here, we began to examine immunotherapy in owned dogs diagnosed with CanL using a new vaccine format, i.e., singlet oxygen-inactivated *Leishmania* promastigotes [15] that was previously found to be immunoprophylactically effective against experimental cutaneous and visceral leishmaniasis in animal models [16,17]. Immunotherapy of diseased dogs with this vaccine format significantly reduced their parasite loads and improved their clinical scores favorably compared to the standard chemotherapy regimens. Odds ratio analysis of the clinical scores further points to the potential benefits of immunotherapy for dogs with prior chemotherapy. Additional data presented further suggests the value of post-therapeutic survival time as a potential criterion to assess vaccine efficacy.

## 2. Materials and Methods

### 2.1. Leishmania Vaccine Preparation for Immunotherapy

Singlet oxygen-killed *Leishmania infantum* was used as the vaccine for immunotherapy. The therapeutic vaccine was prepared from the promastigotes of this species [18], which were fully inactivated with intracellularly generated singlet oxygen, as outlined [15]. Briefly, the transgenic uroporphyrinogenic promastigotes [19] were cultured in Medium 199 plus 10% heat-inactivated fetal bovine serum under drug selection. After growth for one cycle in a drug-free medium to stationary phase, the cells were suspended in Hank’s Balanced Salt Solution with 0.01% bovine serum albumin and rendered urporphyric (URO) cytosolically by exposure in dark to 1 mM delta-aminlevulinate. The transgenic parasites described receive add-on mammalian cDNA transgenes, rendering them responsive to an inducer to accumulate photosensitizers, which produce *Leishmania*-inactivating singlet oxygen intracellularly on exposure to light. These transgenics differ from knockout mutants of *Leishmania* with deletions of their endogenous genes for desirable phenotypes, such as replication arrest. Our transfectants were further loaded endosomally with another photosensitizer by incubation in the presence of 1 µM aminophthalocyanine (PC1) overnight in the dark [20]. The URO/PC1 doubly loaded promastigotes were then exposed to longwave UV and red light, 10 min each, to excite these photosensitizers for the generation of leishmanicidal singlet oxygen in the cytosol and endosome, respectively. The promastigotes so doubly inactivated were verified to remain intact, but non-motile and non-replicative in macrophages, hence producing no lesion in mice [21]. The inactivated promastigotes were resuspended in phosphate-buffered saline at 10^8^ cells/mL and kept at 4 °C or frozen at −20 °C before use for vaccination. The fact that the parasites remain active after freezing–thawing cycle at −20 °C suggests that their vaccine-ability is based on the integrity of their antigens, but not their cellular structural intactness. We hypothesize that the preservation of their antigenic integrity is due to the inhibition of parasite endogenous proteases whose active sites often contain aromatic amino acids, which are susceptible to oxidation specifically by the singlet oxygen generated.

### 2.2. Dogs Enrolled in the Present Study

Male and female dogs of different ages and breeds were enrolled in the present study as they became available at the Department of Veterinary Medicine and Animal Productions, the University of Naples Federico II (Naples, Italy). Dogs with concomitant diseases common in the region, i.e., ehrlichiosis and anaplasmosis caused by other etiological agents, were excluded to avoid their potential interference in the evaluation of treatment response. The diagnosis of these rickettsia diseases was carried out by IFAT and PCR to detect the presence of *Ehrlichia canis* and *Anaplasma* in conjunction with clinical examinations and hematological tests for thrombocytopenia and leukocytosis according to published methods [22].

The enrolled dogs were divided into three groups (Table 1):

**Group 1** included nine dogs for immunotherapy alone. They were diagnosed positive for canine leishmaniasis with overt clinical symptoms and received no treatments before this study. All dogs in this group received immunotherapy with a total of three doses of intradermal vaccination, each with 10^7 1^O_2_-inactivated promastigotes in a volume of 0.1 mL per dog at time 0, week 2, and month 6.

**Group 2** consisted of 14 *Leishmania*-positive and mostly symptomatic dogs for immunotherapy. All dogs of this group had been previously diagnosed positive for leishmaniasis and already received one cycle of chemotherapy with meglumine antimoniate (100 mg/kg/IM/30 days) and Allopurinol (10 mg/kg/SC/day for life) [5] 9–12 months before their enrollment in the present study. All 14 dogs in this group received immunotherapy with dosages and schedules of vaccination exactly as described for Group 1. This group is referred to as Immunotherapy $\bar{P}$ (=after) Chemotherapy.

**Group 3** consisted of 10 dogs, which were diagnosed positive for canine leishmaniasis, but have not received any treatment until the present study. The treatment regimens with meglumine antimoniate were started at time 0 and subsequently with allopurinol, as described above for Group 2 dogs.

The dogs varied considerably among the three groups, since they were enrolled with owners’ consent as they became available, leaving little opportunity to match their breed, sex and age. The average age of the dogs varied by 7–8 months, increasing from Group 1 to Group 3. The number of dogs enrolled in Group 2 (immunotherapy $\bar{P}$ chemotherapy) was higher at 14 than those of Group 1 (immunotherapy alone) and Group 3 (Chemotherapy alone) at 9 and 10, respectively. More dogs were included in Group 2, considering their low parasite loads and clinical scores due to prior chemotherapy.

### 2.3. Post-Therapeutic Clinical and Laboratory Evaluations

All 33 dogs in the three groups were subjected to clinical evaluations and sample collections for laboratory tests at time 0 and then at month 2, 6, 8, and 12. The samples taken included blood for determining hematobiochemical profiles, e.g., blood cell counts and metabolic panels: aspartate transaminase, alanine aminotransferase, total serum proteins, creatinine, urea, albumin/globulin ratio, and immunofluorescence titers of anti-*Leishmania* antibodies (IFAT) as well as saline aspirates of swollen infected lymph nodes to assess parasite loads. The hematobiochemical alterations observed most frequently included moderate anemia, thrombocytopenia, leukopenia, increased β-γ globulins, and hepatic enzymes.

**Clinical scores** were determined on the basis of clinical symptoms/signs and laboratory tests for abnormalities according to the criteria of Solano-Gallego [23]. Each dog was thus assessed for clinical scores on a scale from 0 to 3 as follows:

**Score 0**: Little or no obvious symptoms of leishmaniasis. Negligible titers of anti-*Leishmania* antibodies (IFAT < 1:40).

**Score 1**: Mild disease—Peripheral lymphadenopathy or popular dermatitis; negligible to low titers of anti-*Leishmania* antibodies (IFAT = 1:40); no hematobiochemical abnormalities.

**Score 2**: Moderate disease—Peripheral lymphadenopathy, severe skin involvement, anorexia, weight loss, fever, epistaxis; low to high titers of anti-*Leishmania* antibodies (IFAT = 1:80–1:160); mild hematobiochemical abnormalities.

**Score 3**: Severe disease—Peripheral lymphadenopathy, severe skin involvement, anorexia, weight loss, fever, epistaxis, arthritis, uveitis, glomerulonephritis; medium to high titers of anti-*Leishmania* antibodies (IFAT > 1:160); severe hematobiochemical abnormalities.

***Leishmania* loads** in the popliteal and/or prescapular lymph nodes were determined by real-time (RT) PCR of DNA isolated from the lymph node aspirates, as described previously [22,24]. *Leishmania*-specific DNA was determined by RT Q-PCR analysis using *Leishmania*-specific primer set, i.e., (5′-GCGTTCTGCGAAAACCG-3′, 5′-AAAATGGCATTTTCGGGCC-3′) targeting the conserved region of the *L. infantum* kDNA minicircle (NCBI Accession Number AF291093). The fluorogenic probe was synthesized using an FAM reporter molecule attached to the 5′ end of one primer (5′-AAAATGGCATTTTCGGGCC-3′), and a TAMRA quencher linked to its 3′ end (Applied Biosystems, Foster city, CA, USA). The subsequent steps for RT Q-PCR were carried out as previously detailed, as were the control of host DNA contamination in the aspirate samples and construction of the standard curve of *L. infantum* DNA for extrapolation of the readouts to *Leishmania* cell number/mL [22,24].

All 33 enrolled dogs were monitored for their health status beyond a one-year period of this study during their annual check-ups until their death was notified by the owners and registered in the clinical records for each dog.

### 2.4. Graphics and Statistical Analyses

The values of parasite loads and clinical scores obtained from samples collected at all five post-therapeutic time points were analyzed within each group, but not directly compared among the three groups, given the limited sample sizes (9–14 dogs per group) and substantial variations in their baseline values at time zero across groups. The lymph node parasite load data obtained were subject to Spaghetti plotting to show their changes over time separately for each group and combined for comparison. For each of the three groups, the baseline value of the parasite loads at time 0 was pairwise-compared with each of those obtained at different post-treatment time points for analyses by the Wilcoxon signed-rank test [25].

A Sankey diagram of the clinical scores was created to show their changes over time for all dogs of the individual groups. At each time point, the clinical scores, ranging from 0 (no clinical symptom) to 1–3 (increasing clinical severity), were displayed with increasing intensities of grays to black in stacked bars. The flow of their transition from one time point to the next was shown by connecting ribbons.

To assess changes in symptomatic severity based on the clinical scores in the order of 3 to 0 (severe, moderate, mild, and none) over post-treatment periods, odds ratios of worse versus better clinical symptoms were estimated according to ordinal logistic regression for each group, adjusting for age at the baseline, accounting for the within-subject dependency in scores [26,27]. A random subject effect was included to account for the within-subject correlation of clinical scores [26,27]. This model was fitted using the clmm function from the ordinal R4.3.2 package (version of R4.3.2) [27]. The results allowed us to estimate three probabilities over time of developing: (a) any symptoms during the post-treatment period; (b) moderate to severe symptoms; and (c) severe symptoms for each treatment group. These probabilities were then used to generate graphic presentations for each treatment group.

The post-therapeutic survival time was initially calculated for all 33 dogs in the three groups, each by subtracting their age at the time of their first treatment from the age at their death. A composite graph showing the survival profiles of all three groups was plotted according to the % of dogs that died each year after the initial treatment until no survivors remained. The R 4.3.2 package survival [28,29,30] was used according to the Cox proportional hazard model for pairwise comparisons of post-therapeutic survival times across groups, since this analysis specifically focused on between-group rather than within-group comparisons. The proportional hazards assumption in the Cox model was evaluated using Schoenfeld residuals [31]. Values obtained after adjusting for age, parasite loads, and clinical scores at baseline are presented as such and graphically. An increased risk (risk factor) and a decreased risk (protective factor) are indicated by the values of hazard ratio (HR) of higher and lower than one, respectively.

### 2.5. Ethical Statement

The immunotherapeutic study carried out with the consent of all dog owners was approved by the Internal Evaluation Committee (IEC) of the University of Naples. Dogs diagnosed with leishmaniasis were treated and monitored with the approval of the university Animal Welfare Committee according to the guidelines for the control of canine leishmaniasis legislated by the Regione Campania in southern Italy. The provision requires compulsory medical treatment for such dogs and their periodic monitoring. Written consents from all dog owners were obtained to perform clinical evaluations of the dogs as well as collecting blood and lymph node aspirate samples at the time of diagnosis and during post-therapy follow-ups. All procedures were performed in the presence of the dog owners following good veterinary practice to avoid undue suffering of the dogs. No dog was sacrificed in this study.

## 3. Results

### 3.1. All Dogs Diagnosed Positive for Leishmaniasis Before Therapeutic Treatments

At time 0 immediately before the first therapeutic treatment (Table 2, Column 0), all dogs in the three groups showed positive signs of leishmaniasis based on the values of their parasite loads and/or clinical scores, as assessed by *Leishmania*-specific PCR and the clinical parameters described, respectively. The dogs in Groups 1 and 3 had not received any prior anti-*Leishmania* treatments, hence their parasite loads and clinical scores were generally higher than those of Group 2 previously treated with antimonial chemotherapy. There was a disparity in the readings of parasite loads versus clinical scores among different dogs within each of the three groups. Incongruence in the values between parasite loads and clinical scores was noted for individual dogs.

### 3.2. Post-Therapeutic Assessments of Dogs During the First Year Showed a Downward Trend in Their Disease Progression in All Three Groups

All dogs in the three treatment groups were assessed for their parasite loads and clinical scores at four time points after the initial therapy, i.e., months two, six, eight, and twelve (Table 2, Columns 2, 6, 8, and 12). The values of parasite load and clinical score for the dogs in all three groups trended lower, albeit with spikes in a few cases during the interim periods, e.g., dog #1 of Group 1, dogs # 3, 6, and 14 of Group 2, and dogs # 1, 2, 4, 6, and 8 of Group 3. At the end point of the assessment on the 8th and/or on the 12th month, the dogs of all three groups had significantly diminished parasite loads and clinical scores, where data available, as compared with the starting values at time 0, except for the clinical scores of 3 noted for dogs # 1 and 9 of Group 1 (Table 2, Column 8).

**Figure 1:**
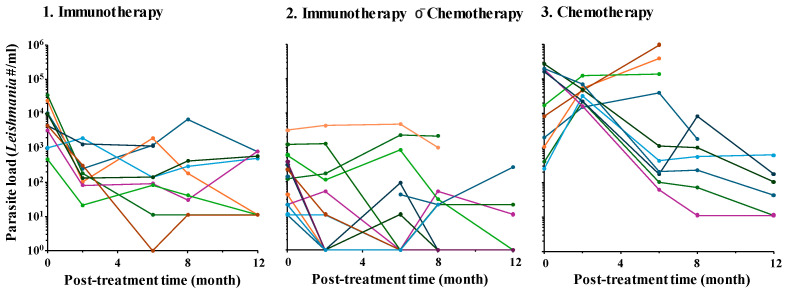
**Spaghetti plot of *Leishmania* loads showing their decrease over post-treatment periods in all three groups.** The values of parasite loads (*Leishmamia* #/mL = parasite number per milliliter) for all dogs in each of the three groups are presented in different colors. **Note:** Excluded are data not available for Group 3 Chemotherapy (see Table 2).

**Figure 2:**
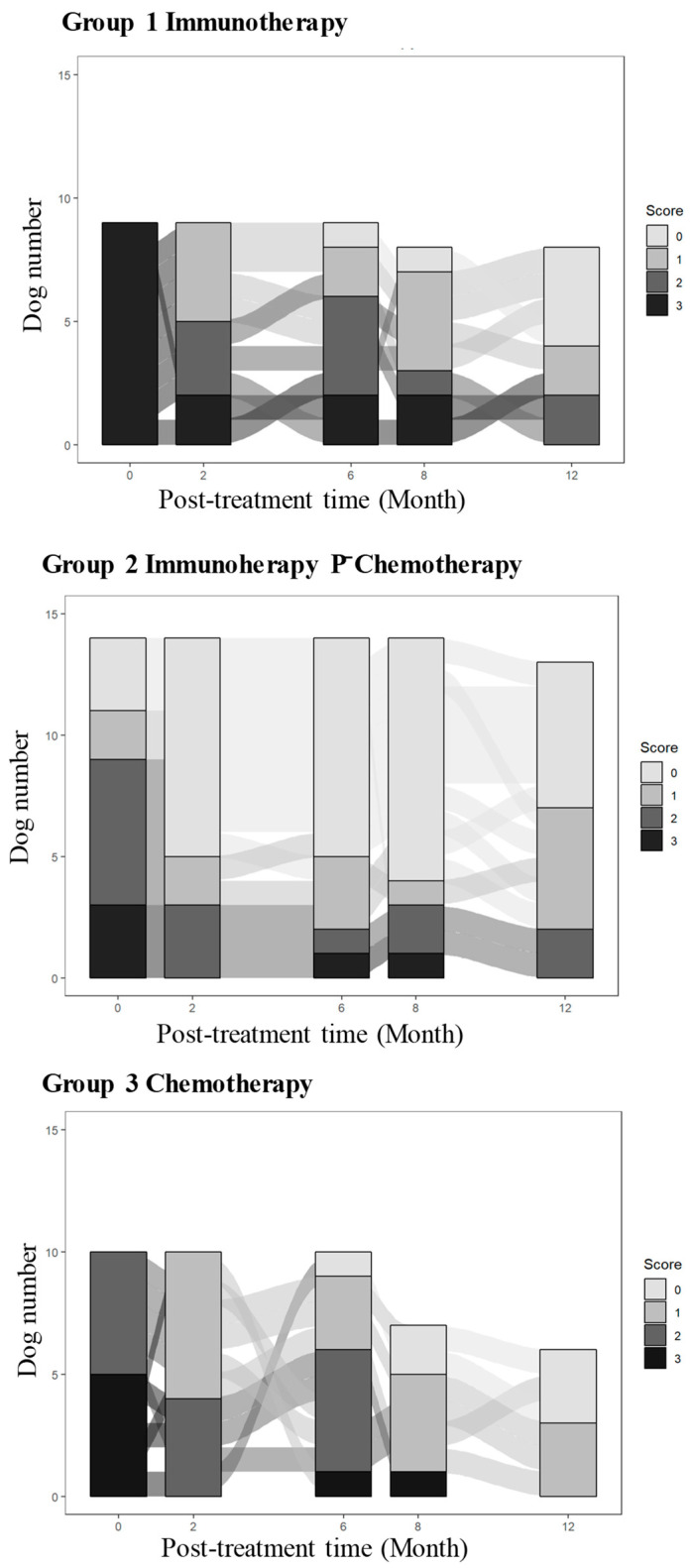
Sankey plots showing clinical scores as stacked bars and the flow of their decrease with time after treatment. Clinical scores of individual dogs in all three groups (Group 1–3) were presented as stacked bars in different shades of gray to black with increasing severity and their changes with time (month 0, 2, 6, 8, and 12) during the one-year period.

**Figure 3:**
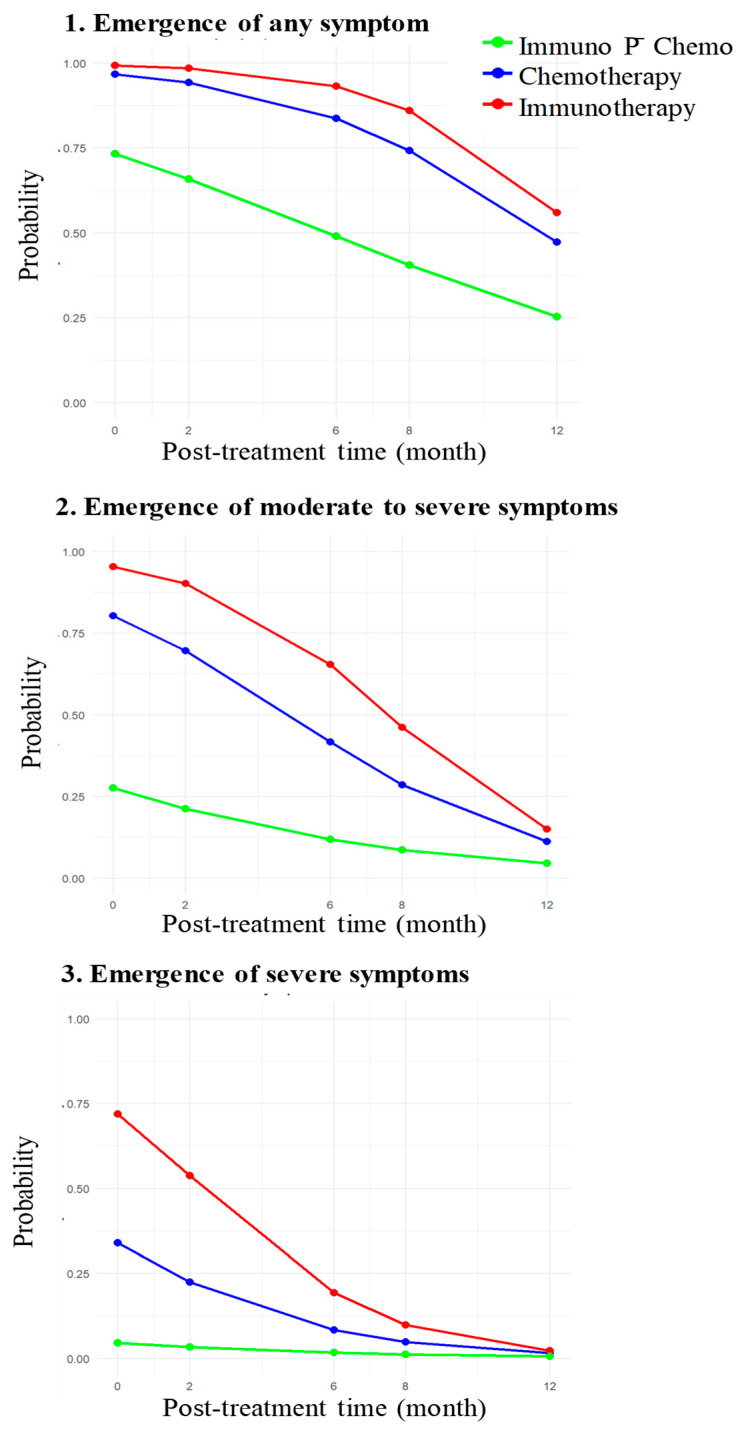
Comparison of the three groups for the emergence of symptomatic severity over time predicted from clinical scores of the dogs. Red, green, and blue lines represent the probabilities of developing various symptoms (1–3) for dogs of the immunotherapy, imuno-chemotherapy and chemotherapy groups, respectively.

Spaghetti plots of parasite load (Figure 1) and Sankey diagrams of the clinical score (Figure 2) of individual dogs in the three groups illustrate the downward trend of both following erratic courses. This is reflected in the group variations seen in the results from statistical analyses of the parasite loads, i.e., pairwise comparisons of the starting value versus those obtained at each subsequent time point within each group. Parasite loads reduced significantly at all time points (*p*-value ≤ 0.01) in Group 1 with immunotherapy alone, at the 8th- and 12th-month time points (*p*-value < 0.05) in Group 2 with immunotherapy $\bar{P}$ chemotherapy and at the 8th-month time point (*p*-value < 0.5) in Group 3 with chemotherapy alone (Table 3). Ordinal logistic regression analysis of the clinical scores predicted the odds ratios for developing worse symptoms in three different scenarios, i.e., (a) any symptoms versus none; (b) moderate-to-severe versus mild-to-none; and (c) severe versus moderate-to-none. The values of odds ratios for all three groups decreased significantly with each unit increase in post-treatment time, with the age of dogs being insignificant, except for those in Group 3 (Table 4). The probability was estimated for developing any symptoms, moderate-to-severe symptoms, and severe symptoms over time for dogs in all three groups, with the lowest being in Group 2 (immunotherapy $\bar{P}$ chemotherapy) (Figure 3).

### 3.3. Post-Therapeutic Survival Time Longer for the Dogs in Groups 1–2 with Immunotherapy Than Those in Group 3 with Chemotherapy Alone

**Figure 4:**
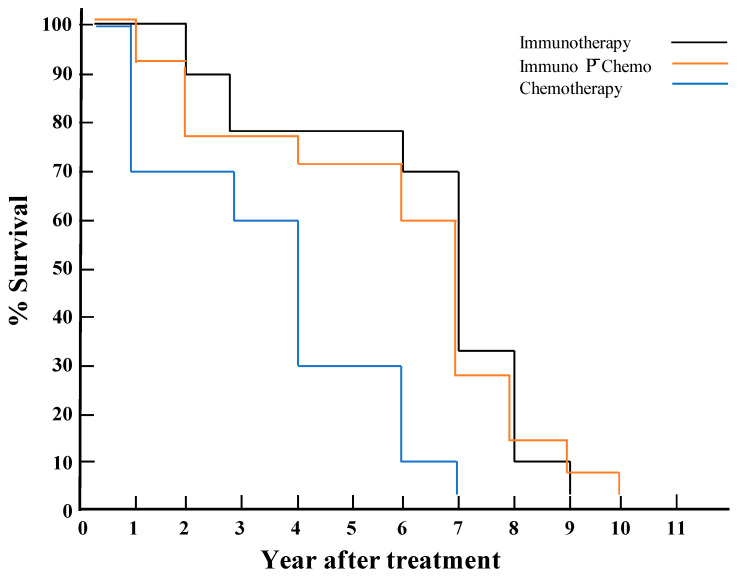
Post-therapeutic survival plot of dogs showing their annual mortality rate in each of the three groups. Black, red, and blue lines represent yearly changes in the annual mortality of dogs in % for immunotherapy, imuno-chemotherapy, and chemotherapy groups, respectively.

**Figure 5:**
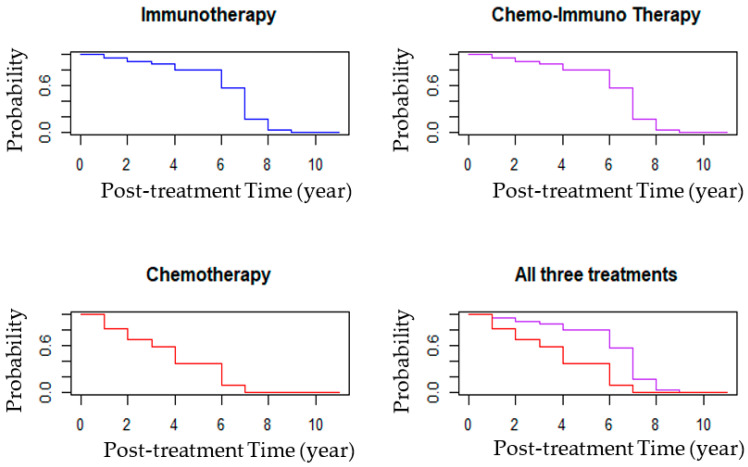
Age-adjusted post-treatment survival plots predicted for the dogs of the three groups according to Cox proportional hazard model. Blue, purple, and red lines represent yearly changes in the values for dogs of the immunotherapy, chemo-immunotherapy, and chemotherapy groups, respectively.

While their parasite loads and clinical scores were not further examined beyond the 1st year after treatment, all dogs of the three groups continued to have annual medical check-up in the clinic until their death. Notably, the average post-therapeutic survival time was 3.7 years for Group 3 dogs in contrast to 6.33 and 6.07 years for Groups 1 and 2, respectively (Table 5). The difference in average post-therapeutic survival time of ≥2.4 years cannot be accounted for by the mean age difference of the dogs at the beginning of the treatments (time 0) between Group 3 and Groups 1–2 (<1.4 year). Post-therapeutic survival plot (Figure 4) based on the yearly mortality rate (%) also showed that more dogs died each year in Group 3 (**Blue line**) than those in Groups 1 (**Black**) and 2 (**Red**), with the yearly death rates of dogs in the latter two Groups being comparable. Consistent with this result was the outcome from pairwise comparisons of the treatment groups based on the Cox proportional hazard model adjusted for age at the baseline (Table 6). The risk of death was significantly lower for dogs in Group 1 (immunotherapy alone) and Group 2 (immunotherapy $\bar{P}$ chemotherapy) compared to those in Group 3 (chemotherapy alone) (*p*-values < 0.03), while this was comparable between Group 1 and Group 2 dogs (*p*-value = 0.99). Additionally, a 60% higher risk of death was found associated with each yearly increase in age from the time of treatment initiation. The estimated survival curves of the dogs in the three treatment groups based on hazard ratios plotted individually and together (Figure 5) were similar in topology to Figure 4.

## 4. Discussion

Evidence presented in the present study shows that immunotherapy of owned dogs in Group 1 with canine leishmaniasis (CanL) reduced their parasite loads and improved their clinical scores. Notably, this effective immunotherapy is achieved by vaccination of dogs, which were in an immunosuppressed state due to the on-going CanL [32]. The present finding is thus different from the previous success using the same vaccine format, i.e., singlet oxygen (^1^O_2_)-killed *Leishmania*, for prophylactic immunization of naive and thus immunologically intact animals against experimental leishmaniasis [16,17]. The immunotherapeutic mechanisms observed are of interest for further investigation. Of relevance to mention are two aspects of the vaccine format used. One is the use of whole-cell *Leishmania*, which has long been recognized as advantageous, as it provides a full complement of all potential vaccine molecules therein. Indeed, live virulent *Leishmania* in small number or those rendered avirulent by cultivation [33] or genetically [34] have been used in leishmanization against human leishmaniasis. Also tried with limited success were *Leishmania* heat-killed by autoclaving or pasteurization plus BCG (see [35]). “Killed” parasites plus nanoparticles were also applied by intranasal sprays [12], or with the addition of saponin by subcutaneous inoculation [36] for immunotherapy of CanL, presenting positive outcomes. Another aspect of interest is the format of the vaccine prepared by rapid and complete inactivation of *Leishmania* via intracellularly generated ^1^O_2_ [15], rendering them not only safe for use but also active, as indicated by antigenicity of their transgenically expressed ovalbumin for epitope-specific T-cell activation [37]. Further investigation is warranted to compare side-by-side different formats of vaccines and different routes of vaccination for immunotherapy of CanL.

Of special interest is our finding of a favorable outcome in Group 2 dogs with CanL, which were subjected to a full cycle of chemotherapy completed 9–12 months before receiving otherwise the same immunotherapy as did the Group 1 dogs. The additional immunotherapy given to Group 2 dogs significantly reduced both parasite loads and clinical scores toward the end, similar to what was observed in Group 1 dogs (immunotherapy alone) and Group 3 dogs (chemotherapy alone). However, according to the calculation of odds ratios by ordinal logistic regression analysis of clinical scores, while dogs in all three groups showed a time-dependent downward trend for the emergence of clinical severity, Group 2 dogs are predicted as the least likely to develop serious clinical symptoms. This prediction is supported by the odds ratios across all three worse-versus-better scenarios, lending credence to the validity of the prediction. Clearly, the prior chemotherapy of the Group 2 dogs accounts for their better performance compared to the Group 1 dogs, since that is the known treatment difference between them. The simplest explanation is an additive or cumulative effect of immunotherapy plus prior chemotherapy in Group 2 dogs. Their relatively low parasite loads and clinical scores at the beginning of immunotherapy may be taken to support this assumption, notwithstanding the long time-lag of 9–12 months between the two treatments for this group of dogs. A more nuanced explanation may be considered in line with the generally accepted notion that chemotherapeutic cure of all infectious diseases ultimately depends on the immune clearance of the causative agents, since no drug is expected to reach all intended targets in a given subject to account for their elimination. If so, antigens released from the parasites killed by prior chemotherapy of the Group 2 dogs elicit immunity, which may however be insufficiently robust to suppress parasites for effective mitigation of the clinical symptoms. The subsequent immunotherapy of the Group 2 dogs may have strengthened the pre-existing drug-induced feeble immunity, accounting for their improved odds of not suffering from severe clinical outcomes. In leishmaniasis, including CanL, the inability of chemotherapy to elicit effective immune clearance of *Leishmania* may result in part from their residence in macrophages of the host—an intracellular site difficult to reach by drug unless encapsulated in liposomes [38]. In any event, consecutive treatments of CanL with chemotherapy followed by immunotherapy may be advantageous to alleviate clinical symptoms, as suggested by the findings of this study. The advantages of chemo-immunotherapy for visceral leishmaniasis are evident, as discussed in a review on this subject [39].

The post-therapeutic survival time of dogs was examined in the present study, probably for the first time. This is a fortuitous finding made possible by the availability of records for the year of death of the dogs under investigation. Of particular interest are the post-therapeutic survival plots, showing that Group 3 dogs receiving chemotherapy alone died progressively sooner than those in Groups 1 and 2 receiving immunotherapy. The outcome is similar when the risk of death among the three groups of dogs was assessed by age-adjusted pairwise comparisons of their clinical scores based on the hazard ratios determined according to the Cox proportional hazard model. The congruence of the two data sets for the post-therapeutic survival strengthens the observation. Since an autopsy of the dogs was not performed, the cause of their death is not precisely known. The role of CanL cannot be ruled out, especially in Group 3 dogs starting with high baseline readings of parasite loads and clinical scores. The relevance of these phenotypes to the post-therapeutic survival time of the dogs is however enigmatic, as the cause of death in all visceral leishmaniasis is due to a variety of secondary effects in response to dysfunctional immune responses [40], e.g., kidney failure, anemia, cancer and secondary infection. How immunotherapy prolongs the survival of the diseased dogs more favorably than chemotherapy deserves further investigation. The data presented indicate post-therapeutic survival time as a potential additional criterion useful to assess the therapeutic efficacy for CanL.

## 5. Concluding Remarks

There are two major contributions of the work presented. One is the preliminary finding of the immunotherapeutic potential of a new vaccine format against CanL. Another is the use of odds ratio and hazard ratio models for clinical data analysis to strengthen the interpretations of the positive, albeit more scattered results, as expected from a trial by enrolling a small number of diseased dogs (average number = 10). Such direct observational study has the cost-effective expediency at the expense of extensive considerations of inclusive/exclusive criteria. As such, it represents a vaccine test in the “real world”. Alternatively, the outcome may be considered as a prelude to a formal costly vaccine clinical trial, for which a larger sample size predetermined by power analysis statistics is needed to incorporate a range of biological variables as covariates to avoid bias, for example, in age, sex, and breed among the different groups; see ref. [41]. The future direction of research to exploit canine immunity for immunotherapy (and immunoprophylaxis) of CanL is expected to follow the same approach mentioned for human leishmaniasis [42]. Specifically, development of canine pluripotent stem cells [43] into organoids of relevance to Can-L is desirable for the study of infection and immunity in vitro. While the development of human lymphoid organoids and the introduction of immune cells into organoids via vasculature are still under study, a provisional approach to study human cancer immunotherapy in vitro was developed by using tumor organoids (or tissue explants) flooded with immune cells from the blood or lymphoid organs from the same individual [44]. Adaptation of such an approach may mitigate some obvious limitations of the present investigation. Foremost is its potential to address the variability in individual dogs in response to the therapeutics due to their differences in sex, age, and breed. In vitro organoid models have the advantages of the ease to set up equal baseline levels of infection among the treatment groups for fair comparisons, and to facilitate more frequent and repeated sampling, thereby increasing the accuracy in assessments of the outcome. In addition, untreated controls can be set up in vitro as a reference for comparing the relative efficacy of different treatments. Clearly, the in vitro model as mentioned is suitable to address many specific limitations encountered here in the in vivo work with dogs, e.g., unequal starting baselines, precluding fair comparison among the treatment groups; unexpected erratic spikes of parasite load during the post-therapeutic period; incongruity of parasite loads versus clinical scores; and the inability to set up a control group of untreated dogs due to the mandatory requirement to treat all diseased dogs. Development of canine organoid model for CanL also has the potential for CAR T immune cell therapy along the line of individualized or precision medicine.

## Figures and Tables

**Table 1:** Owned dogs enrolled into three groups for different therapeutic treatments.

**Group 1: Immunotherapy**			
**Dog**	**Breed**	**Sex**	**Age (Yr)**
**1**	Mongrel	M	8
**2**	Mongrel	F	5
**3**	Setter	F	8
**4**	Mongrel	M	6
**5**	Mongrel	M	7
**6**	Setter	F	3
**7**	Coker	M	5
**8**	Mongrel	F	6
**9**	Breton	M	5
			**Mean = 5.9**
**Group 2: Immunotherapy $\bar{P}$ Chemotherapy**			
**Dog**	**Breed**	**Sex**	**Age (Yr)**
**1**	Jack Russel	M	9
**2**	Setter	M	11
**3**	Boxer	F	9
**4**	Coker	F	6
**5**	Setter	M	3
**6**	Pointer	M	5
**7**	Setter	M	7
**8**	Setter	M	7
**9**	Kurzhaar	M	4
**10**	Jack Russel	M	7
**11**	Setter	F	4
**12**	Setter	F	5
**13**	Hunting dog	M	3
**14**	Mongrel	M	9
			**Mean = 6.4**
**Group 3: Chemotherapy**			
**Dog**	**Breed**	**Sex**	**Age (Yr)**
**1**	Doberman	M	7
**2**	German Shepherd	M	8
**3**	Setter	M	7
**4**	Setter	M	7
**5**	Boxer	F	9
**6**	Mongrel	M	10
**7**	Husky	M	9
**8**	Mongrel	F	6
**9**	Breton	F	5
**10**	German Shepherd	M	5
			**Mean = 7.3**

**Table 2:** Lymphnode parasite loads/clinical scores of the dogs assessed at different times post-therapeutic treatments.

Parasite #/mL (×10^−3^)/Clinical Score Post-Treatment Time in Month:					
	0	2	6	8	12
**Group 1: Immunotherapy**					
**Dog#**					
**1**	10.4/3	0.25/1	1.20/2	6.84/3	0.78/2
**2**	24.0/3	0.10/2	1.90/2	0.18/1	0.01/0
**3**	34.9/3	0.18/3	0.01/2	0.01/0	0.01/0
**4**	01.0/3	01.9/1	0.14/2	0.29/1	0.50/1
**5**	3.20/3	0.08/2	0.09/1	0.03/2	0.80/2
**6**	0.47/3	0.02/1	0.08/1	0.04/1	0.01/0
**7**	4.53/3	1.29/3	1.12/3	#--	#--
**8**	4.41/3	0.31/1	0.00/0	0.01/1	0.01/0
**9**	10.0/3	0.13/2	0.14/3	0.41/3	0.57/1
**Group 2: Immunotherapy $\bar{P}$ Chemotherapy**					
**Dog#**					
**1**	0.01/1	0.00/0	0.00/0	0.00/0	0.00/0
**2**	0.04/2	0.00/0	0.00/0	0.00/0	0.00/0
**3**	0.12/3	0.17/2	2.28/2	2.11/2	#--/2
**4**	0.01/0	0.01/0	0.00/0	0.00/0	0.00/1
**5**	0.02/1	0.05/0	0.00/0	0.05/0	0.01/0
**6**	0.59/2	0.11/1	0.85/1	0.03/0	0.00/0
**7**	0.31/2	0.00/0	0.09/1	0.00/1	0.00/1
**8**	0.22/2	0.01/1	0.00/0	0.00/0	0.00/1
**9**	0.02/0	0.00/0	0.01/0	0.00/0	0.00/0
**10**	0.14/2	#--/0	0.04/0	0.02/0	0.26/1
**11**	0.38/2	0.00/0	0.00/0	0.00/0	0.00/0
**12**	1.19/3	1.25/2	0.00/1	0.02/0	0.02/1
**13**	0.02/0	0.00/0	0.00/0	0.02/3	#--
**14**	3.18/3	4.33/2	4.69/3	0.97/2	#--/2
**Group 3: Chemotherapy**					
**Dog#**					
**1**	1.96/3	15.15/2	39.89/2	1.80/3	#--
**2**	1.06/3	53.0/1	391.0/3	#--	#--
**3**	0.39/2	22.1/1	0.10/2	0.07/1	0.01/1
**4**	0.24/2	31.1/1	0.41/1	0.54/1	0.61/1
**5**	185.0/3	16.00/1	0.06/1	0.01/1	0.01/1
**6**	16.77/3	125.0/2	137.7/2	#--	#--
**7**	158.2/2	22.00/2	0.17/2	8.12/1	0.17/0
**8**	8.33/2	46.56/1	967.7/2	#--	#--
**9**	26.41/2	48.0/1	1.10/1	1.00/0	0.10/0
**10**	195.3/3	69.1/2	0.20/0	0.22/0	0.04/0

**#**, Data not available.

**Table 3:** Statistical analysis of parasite loads from time 0 versus each of the post-treatment time points.

Post-Treatment in Month:					
0		2	6	8	12
**Group 1: Immunotherapy**					
* Median	4.53	0.180	0.140	0.110	0.255
**† *p* value**	-	**0.0117**	**0.0039**	**0.0078**	**0.0078**
**Group 2: Immunotherapy $\bar{P}$ Chemotherapy**					
* Median	0.13	0.010	0.000	0.010	0.000
**† *p* value**	-	0.4580	0.3457	**0.0426**	**0.0182**
**Group 3: Chemotherapy**					
* Median	12.55	38.830	0.755	0.540	0.0700
**† *p* value**	-	1.0000	0.9219	**0.04688**	0.0625

* From the values of parasite loads Parasite #/mL (× 10^−3^) of 9, 14, and 10 dogs for Groups 1–3, respectively, except where data were not available (see Table 2). † From pairwise comparison of the median values for parasite loads at time 0 versus those of months 2–12, respectively (see Methods section for Wilcox signed rank test). **Note:** Significant *p* values in **bold face**.

**Table 4:** Ordinal logistic regression analysis of the clinical scores to predict odds ratio of symptomatic severity (worse vs. better) for the three groups of dogs with different therapeutic treatment.

	Odds Ratio	95% CI	*p* Value
**Group 1: Immunotherapy (9 dogs)**			
**^1^ Time**	0.7002	(0.5874, 0.8346)	<0.0001
**^2^ Age**	1.2074	(0.7526, 1.9373)	0.435
**Group 2: Immunotherapy $\bar{P}$ Chemotherapy (14 dogs)**			
**Time**	0.8418	(0.7377, 0.9607)	0.0106
**Age**	0.9013	(0.5904, 1.3759)	0.6302
**Group 3: Chemotherapy (10 dogs)**			
**Time**	0.6759	(0.5602, 0.8155)	<0.0001
**Age**	1.5622	(1.0719, 2.2769)	0.0203

^1^ Post-therapeutic time; ^2^ Baseline age at time 0 of treatment.

**Table 5:** Post-therapeutic survival years of individual dogs in the three groups.

**Group 1: Immunotherapy**			
**Dog#**	**Age of Treatment**	**Year of Death**	**Survival Time (Yr)**
**1**	8	10	2
**2**	5	8	3
**3**	8	14	6
**4**	6	13	7
**5**	7	14	7
**6**	3	10	7
**7**	5	13	8
**8**	6	14	8
**9**	5	14	9
			**Mean ± SD = 6.33 ± 2.35**
**Group 2: Immunotherapy $\bar{P}$ Chemotherapy**			
**Dog#**	**Age of Treatment**	**Year of Death**	**Survival Time (Yr)**
**1**	9	10	1
**2**	9	11	2
**3**	11	13	2
**4**	9	13	4
**5**	6	12	6
**6**	3	9	6
**7**	7	14	7
**8**	7	14	7
**9**	7	14	7
**10**	6	13	7
**11**	4	12	8
**12**	5	13	8
**13**	5	14	9
**14**	3	14	11
			**Mean ± SD = 6.07 ± 2.87**
**Group 3: Chemotherapy**			
**Dog#**	**Age of Treatment**	**Year of Death**	**Survival Time (Yr)**
**1**	7	8	1
**2**	8	9	1
**3**	7	8	1
**4**	7	10	3
**5**	9	13	4
**6**	10	14	4
**7**	9	13	4
**8**	6	12	6
**9**	5	11	6
**10**	5	12	7
			**Mean ± SD = 3.7 ± 2.21**

**Table 6:** Risk of death for dogs in the three treatment groups predicted from hazard ratios determined by pairwise comparisons of their clinical scores according to the Cox model and its age-independence.

Groups Paired for Comparison	Hazard Ratio	Coefficient	SE	*p* Value
Immunotherapy vs. Chemotherapy	0.23	−1.47	0.63	0.0203
Immuno $\bar{P}$ Chemo vs. Chemotherapy	0.23	−1.48	0.63	0.0186
Immuno $\bar{P}$ Chemo vs. Immunotherapy	0.99	−0.01	0.55	0.9873
* Age	1.63	0.49	0.13	0.0014
* Parasite load	0.99	−0.01	0.01	0.2078
* Clinical score	0.98	−0.03	0.22	0.9096

* Hazard Ratio (HR): >1 = Increased risk (risk factor); <1 = decreased risk (protective factor).

## Data Availability

All data available are presented in this article.
